# An ATP and Oxalate Generating Variant Tricarboxylic Acid Cycle Counters Aluminum Toxicity in *Pseudomonas fluorescens*

**DOI:** 10.1371/journal.pone.0007344

**Published:** 2009-10-07

**Authors:** Ranji Singh, Joseph Lemire, Ryan J. Mailloux, Daniel Chénier, Robert Hamel, Vasu D. Appanna

**Affiliations:** Department of Chemistry and Biochemistry, Laurentian University, Sudbury, Ontario, Canada; University of Texas-Houston Medical School, United States of America

## Abstract

Although the tricarboxylic acid (TCA) cycle is essential in almost all aerobic organisms, its precise modulation and integration in global cellular metabolism is not fully understood. Here, we report on an alternative TCA cycle uniquely aimed at generating ATP and oxalate, two metabolites critical for the survival of *Pseudomonas fluorescens*. The upregulation of isocitrate lyase (ICL) and acylating glyoxylate dehydrogenase (AGODH) led to the enhanced synthesis of oxalate, a dicarboxylic acid involved in the immobilization of aluminum (Al). The increased activity of succinyl-CoA synthetase (SCS) and oxalate CoA-transferase (OCT) in the Al-stressed cells afforded an effective route to ATP synthesis from oxalyl-CoA via substrate level phosphorylation. This modified TCA cycle with diminished efficacy in NADH production and decreased CO_2_-evolving capacity, orchestrates the synthesis of oxalate, NADPH, and ATP, ingredients pivotal to the survival of *P. fluorescens* in an Al environment. The channeling of succinyl-CoA towards ATP formation may be an important function of the TCA cycle during anaerobiosis, Fe starvation and O_2_-limited conditions.

## Introduction

The TCA cycle is a universal metabolic network in all aerobic organisms. In its catabolic role, this metabolic engine essentially oxidizes acetyl-CoA into CO_2_ with the concomitant production of NADH, FADH_2_ and nucleoside triphosphates by substrate-level phosphorylation. The NADH and FADH_2_ provide the reductive power to generate a proton motive force that is eventually converted into ATP via oxidative phosphorylation [1]. The eight enzymes that constitute this metabolic pathway work in a synergistic fashion to oxidize acetyl-CoA. In its anabolic function, the TCA cycle helps produce α-ketoglutarate, oxaloacetate, succinyl-CoA, and succinate, metabolites that are vital for the production of non-essential amino acids, fatty acids and heme respectively [2], [3].

However, numerous organisms have also evolved to utilize various modified versions of the TCA cycle in order to survive extreme conditions or produce unique metabolites. Some autotrophic organisms for example invoke a reductive TCA cycle to fix CO_2_ into acetyl-CoA [4]. This is in essence a reverse oxidative TCA cycle. In this instance, two molecules of CO_2_ are converted into one molecule of acetyl-CoA. The key enzymes ATP-citrate lyase, fumarate reductase, and α-ketoglutarate ferredoxin oxidoreductase differentiate this metabolic module from its oxidative counterpart. An alternative TCA cycle has been shown to promote the survival of organisms proliferating in dicarboxylic acids even in the absence of α-ketoglutarate dehydrogenase (KGDH). In this situation, α-ketoglutarate decarboxylase coupled with succinate semialdehyde dehydrogenase enables the organism to generate reducing equivalents [5]. Modified TCA cycles also appear to be critical in the adaptation to a toxic environment. We have recently identified a modified TCA cycle with decreases in NAD-dependent isocitrate dehydrogenase (NAD-ICDH) and KGDH activities and an increased NADP-dependent isocitrate dehydrogenase (NADP-ICDH) activity [6]. This alternative TCA cycle plays a critical role in the adaptation of the cellular systems to oxidative stress owing to its ability to produce increased amounts of the antioxidant NADPH and decreased amounts of the pro-oxidant NADH. Furthermore, α-ketoglutarate (KG) also contributes to the detoxification of ROS [6]. The tailoring of the TCA cycle towards an antioxidant defense network appears to be orchestrated by NAD kinase and NADP phosphatase [7].

Although many aspects of this metabolic machinery have been delineated, its regulation, its interaction with other metabolic pathways, the significance of its non-cyclic attributes and its integration into the global molecular networks have yet to be fully elucidated. In this report, we have identified an alternative TCA cycle that enables the soil microbe *P.fluorescens* exposed to Al to generate ATP by a substrate-level phosphorylation module as oxidative phosphorylation, a process reliant on iron (Fe) is severely impeded. Al promotes dysfunctional Fe metabolism [8]. This novel ATP-producing module works in tandem with AGODH, SCS, OCT, and ICL to generate oxalate, an Al-sequester. The diversion of succinyl-CoA toward ATP synthesis during Al-toxicity, Fe-deprivation, and anaerobiosis is also discussed.

## Materials and Methods

### Microbial growth conditions and cellular fractionation

*Pseudomonas fluorescens* (ATCC 13525) was obtained from the American Type Culture Collection. The microbe was maintained and grown in a mineral medium consisting of Na_2_HPO_4_ (6 g), KH_2_PO_4_ (3 g), NH_4_Cl (0.8 g), MgSO_4_.7H_2_O (0.2 g), and 19 mM citrate as the sole carbon source. Trace elements were present in concentrations as described previously [9]. In the Al-stressed medium, the citric acid was complexed to AlCl_3_▪6H_2_O (15 mM). Control cells were grown in citrate alone. The pH was adjusted to 6.8 with 1N NaOH and then autoclaved. The media was dispensed in 200 mL aliquots in 500 mL Erlenmeyer flasks and inoculated with 1 mL of stationary phase cells grown in a citrate (control) medium. The cultures were aerated on a gyratory water bath shaker (model G76, New Brunswick Scientific) at 26°C and 140 rpm. Bacterial cells were isolated by centrifugation at similar growth phases (24 h for control and 28 h for Al-stressed) and then re-suspended in a cell storage buffer (CSB) consisting of 50 mM Tris-HCl, 5 mM MgCl_2_, 1 mM PMSF (pH 7.3). Membrane and soluble fractions were isolated as described previously [8]. Purity of each fraction was tested by measuring the activities of glucose-6-phosphate dehydrogenase (soluble enzyme) and succinate dehydrogenase (membrane-bound enzyme). These cell free extract (CFE) fractions were kept at 4°C for up to 5 days and the various enzymatic activities were monitored. The concentration of protein in each CFE fraction was determined using the Bradford assay [10]. Bovine serum albumin served as the standard.

### Enzymatic assays

The activities of several membrane-bound and soluble enzymes (0.2–0.4 µg protein equivalent/mL) were monitored using an Ultraspec Pro 3100 spectrophotometer. The activities of aconitase (ACN) and fumarase (FUM) were detected by monitoring the formation of the cis-aconitate and the disappearance of fumarate at 220 nm, respectively [8]. α-Ketoglutarate dehydrogenase (KGDH), NAD-dependent isocitrate dehydrogenase (NAD-ICDH), malate synthase (MS), and isocitrate lyase (ICL) activities were assessed at 450 nm using the 2,4-dinitrophenylhydrazine assay (DNPH) as described in [11]. While the formation of α-ketoglutarate (KG) was monitored in the case of NAD-ICDH and ICL, the utilization of the substrates, glyoxylate and KG were assessed for MS and KGDH respectively. The activity of succinate dehydrogenase (SDH) was probed using 2,6-dichloroindophenol (DCIP) as the chromophore [12]. The disappearance of the blue colour due to the donation of electrons from succinate to DCIP was monitored at 600 nm. OCT was monitored by coupling the activity of this enzyme to DCIP and exogenous SDH. Control membrane fraction (0.4 mg protein equivalent/mL) served as the exogenous source of SDH. The control membrane CFE served as an exogenous source of SDH. SCS activity was monitored using the 5,5′-dinitrothiobenzoic acid (DTNB) assay [13]. The interaction of DTNB with CoA was assessed at 412 nm. While citrate synthase (CS) was measured with the DTNB assay, malate dehydrogenase (MDH) was monitored via the production of NADH in the presence of malate [14].

### Heme measurement

The heme assay was performed as described in [15]. A 0.1 mg protein equivalent of membrane fraction was treated with 200 µL of 5% (v/v) Triton X-100 (in pure methanol) and the volume was adjusted to 1 mL with 0.1N NaOH. Following a 0 min incubation at room temperature, the precipitate was removed by centrifugation and the supernatant was subjected to spectrophotometric analysis. Soluble fraction was treated in a similar fashion except a 0.5 mg/mL equivalent of protein was used and the centrifugation step was not necessary. Heme was monitored at 405 nm. The assay was standardized using bovine hemoglobin.

### Blue Native Polyacrylamide Gel Electrophoresis (BN PAGE) and two-dimensional (2D) SDS-PAGE

Blue Native polyacrylamide gel electrophoresis (BN PAGE) and in-gel activity stains were performed as described previously [16]. Membrane proteins were prepared in blue native (BN) buffer (50 mM Bis-Tris, 500 mM 6-aminohexanoic acid, pH 7.0, 4°C) and 1% β-dodecyl-D-maltoside. The in-gel visualization of enzyme activity was ascertained by coupling the formation of NADH/NADPH to 0.3 mg/mL of phenazine methosulfate (PMS) and 0.5 mg/mL of iodonitrotetrazolium (INT). AGODH was visualized using 5 mM glyoxylate, 0.66 mM CoA, 0.83 mM NADP, INT, PMS and reaction buffer. The in-gel activity of Complex I was probed using 5 mM NADH, INT, and reaction buffer containing 5 mM KCN. Complex IV was tested using cytochrome C and diaminobenzidene [17]. SCS was detected by adding 1 mM succinyl-CoA, and 0.1 mM ADP. The formation of ATP was coupled to 5 mM glucose, 5 units of hexokinase, 5 units of glucose-6-phosphate, 5 units of glucose-6-phosphate dehydrogenase, 0.5 mM NADP, INT, PMS, and reaction buffer [16]. OCT activity was ascertained by adding 1 mM succinyl-CoA, 1 mM oxalate, 15 µg of protein equivalent from *P.fluorescens* membrane fraction (which contains SDH), INT, PMS, and reaction buffer. Reactions were stopped using destaining solution (40% methanol, 10% glacial acetic acid) once the activity bands reached their desired intensity. Appropriate controls involving the omission of substrates and enzymes were utilized. Bands were quantified using SCION imaging software for Windows. The resulting bands were documented and used for 2D SDS-PAGE analysis.

2D SDS-PAGE gels were performed in accordance with the modified method described in [18]. Activity bands from blue native gels were precision cut from the gel and incubated in denaturing buffer (1% β-mercaptoethanol, 5% SDS) for 30 min, and then loaded vertically into the SDS gel. SDS-gels (10% isocratic) were favoured for the proper separation of protein. Kaleidoscope standards (Bio-Rad) were used to identify the molecular mass (MM) of the proteins. Proteins were detected by staining the gel with silver staining.

### Metabolite analysis

The cellular levels of various metabolic intermediates and nucleotides were assessed by HPLC. Briefly, a 2 mg protein equivalent to soluble CFE from control and Al-stressed cells (isolated at 24 h and 28 h, respectively) was boiled for 2 min and then injected into an Alliance HPLC equipped with a C18-reverse phase column (Synergi Hydro-RP; 4 µm; 250×4.6 mm, Phenomenex) operating at a flow rate of 0.7 mL/min and 26°C. The proper separation of the metabolites was achieved using a mobile phase consisting of 20 mM K_2_HPO_4_ (pH 2.9) [7], [19]. The ability of glyoxylate to produce ATP by substrate level phosphorylation was assessed by incubating 2 mg of protein equivalent to the CFE in a phosphate reaction buffer (20 mM NaH_2_PO_4_, 5 mM MgCl_2_, pH 7.4) containing 5 mM succinate, 5 mM glyoxylate, 0.5 mM CoA, 2 mM ADP, and 0.5 mM NADP for 1 h in the presence of 5 mM KCN. The latter inhibits complex IV. The use of succinyl-CoA for ATP production was monitored by incubating 0.2 mg membrane protein in a phosphate reaction buffer containing 1 mM succinyl-CoA and 1 mM ADP for 1 h. To ascertain whether ATP was preferentially being generated by substrate-level phosphorylation, the CFE from the control and stressed cultures was incubated with 5 mM citrate, 0.5 mM ADP, 0.5 mM NADP, and 0.1 mM CoA in the absence and presence of 5 mM KCN. The activity of OCT was tested by monitoring the transfer of CoA from succinyl-CoA to oxalate. CFE (2 mg of protein equivalent) in a phosphate reaction buffer containing 0.1 mM succinyl-CoA and 0.1 mM oxalate was reacted for 5 min then quenched for HPLC as described above. Organic acids (oxalate, glyoxylate, and succinate) and nucleotides (ATP, ADP, oxalyl-CoA, and succinyl-CoA) were monitored at 210 nm and 254 nm, respectively. Negative reactions were performed in the absence of substrates. The retention time of all metabolites was confirmed by injecting known standards at various concentrations. The levels of the respective metabolites were quantified using standard curves and the EMPOWER software. Oxalyl-CoA was also identified by HPLC. Following the 15 min reaction, the CFE (0.2 mg protein/mL) with 0.5 mM succinyl-CoA and 1 mM oxalate, an unidentifiable peak at 10 min was collected and then treated with 6N KOH for 1 h [20]. The hydrolytic digest was then monitored at 254 nm for CoA and 210 nm for oxalate. Oxalate and CoA were identified by injecting known standards.

^13^C-NMR analyses were performed using a Varian Gemini 2000 spectrometer operating at 50.38 MHz for ^13^C. Samples were analyzed with a 5 mm dual probe (35° pulse, 1-s relaxation delay, 8 kilobytes of data, and 2000 scans). Chemical shifts were referenced to standard compounds under analogous conditions. The formation of succinyl-CoA was probed by incubating a 2 mg protein equivalent of control or Al-stressed CFE for 1 h in a phosphate reaction buffer containing [1,4-^13^C_2_] succinate, 5 mM glyoxylate, 0.5 mM CoA, 2 mM ADP, and 0.5 mM NADP. Metabolites were identified by comparing to known standards. Reactions were quenched by boiling and then subjected to spectral analysis.

### Regulation of AGODH activity

The Al-mediated modulation of AGODH activity was further delineated by performing regulation experiments. Control cells (10 mg protein equivalent) grown in citrate for 24 h were transferred to an Al-containing medium. Following an 18 h exposure, the cells were harvested and treated for BN PAGE as described above. Similar experiments were performed with Al-stressed cells (grown for 28 h) transferred into a control medium.

### Statistical analysis

Data were expressed as means±standard deviations. Statistical correlations of data were checked for significance using the Student t test (p≤0.05). All experiments were performed at least twice and in triplicate.

## Results

### Al toxicity perturbs aerobic respiration in P. fluorescens

**Figure 1:**
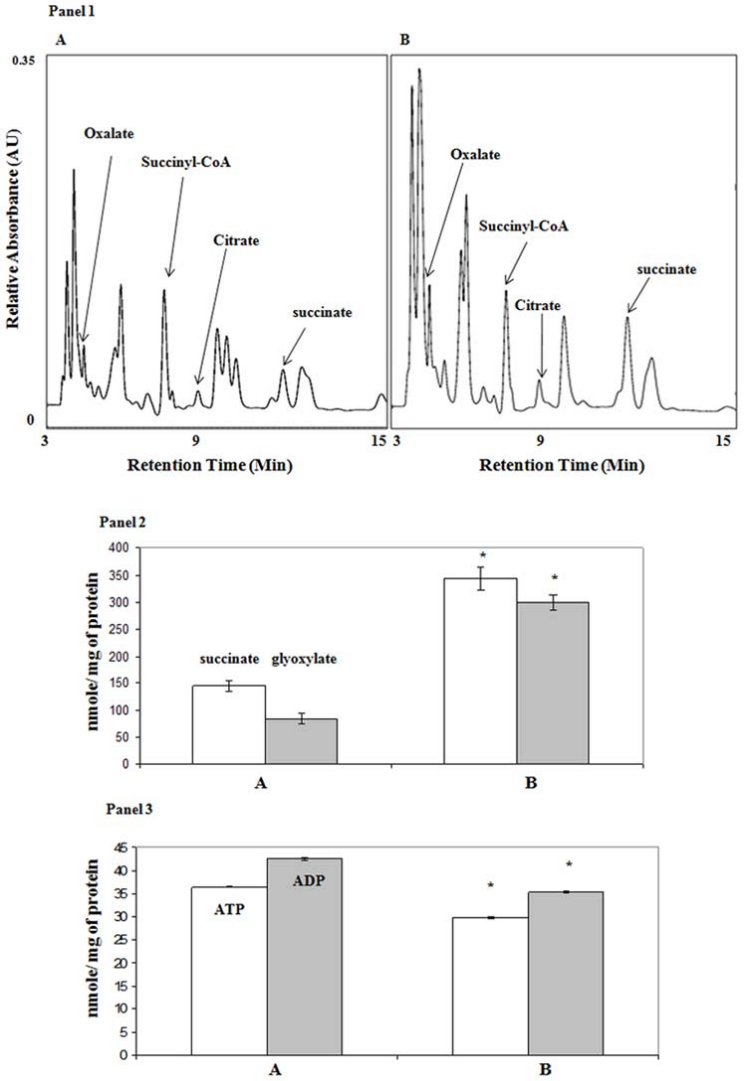
HPLC analysis of targeted metabolites in *Pseudomonas fluorescens*. Panel 1: Soluble CFE was utilized A) control (citrate) cultures and B) Al-stressed (Al-citrate) cultures. Panel 2: Succinate and glyoxylate levels in *P. fluorescens* exposed to A) control B) Al-stressed conditions. White bars  =  succinate, Gray bars  =  glyoxylate. Panel 3: ATP and ADP levels in *P. fluorescens* exposed to A) control B) Al-stressed conditions. White bars  =  ATP, gray bars  =  ADP. n = 3, p≤0.05, mean±S.D. * represents statistical significance in comparison to control. Cells were isolated at similar growth intervals to afford a proper comparison (control  = 24 h, Al-stress  = 28 h). Peaks were quantified using EMPOWER software and identified by injecting known standards.

In order to further delineate the molecular mechanisms involved in the adaptation of *P. fluorescens* to Al toxicity, the metabolite profile from the CFE of the control and Al-stressed cells were analyzed. Marked variations in such metabolites as citrate, oxalate, and succinate were observed (Figure 1, Panel 1). While the level of glyoxylate was sharply increased in the Al-stressed cells, there was not a pronounced variation in the total amounts of ATP (Figure 1, Panel 2,3). To probe the nature of this disparate metabolic profile further, the specific activities of several TCA cycle enzymes and respiratory complexes were assessed. The activities of ACN, KGDH, NAD-ICDH, and SDH were all severely affected in the Al-stressed cells (Table 1). In addition, complex I and IV, two key enzymes in the respiratory chain displayed a significant reduction in activity (Table 1). No discernable variation in the activity of CS and MDH was observed. Despite the marked decrease of the key enzymes involved in oxidative phosphorylation, the ATP level in the Al-stressed cells did not undergo a corresponding reduction. Hence, it became evident that the cells challenged with Al were fulfilling their energy needs via an alternative mechanism.

**Table 1:** The relative activity of selected TCA cycle and ETC enzymes in the presence of Al.

Enzyme	Al-stressed activity
ACN^a^	37%
NAD-ICDH^b^	68%
KGDH^b^	32%
SDH^c^	29%
FUM^a^	108%
Complex I^d^	72%
Complex IV^d^	Negligible
ICL^b^	464%
MS^b^	115%

All activities were expressed as percent of control.

**a:** double bonds were measured at 220 nm. 100% corresponds to 124±10 nmol x min^−1^ mg protein^−1^ for ACN and 3.0±0.2 nmol x min^−1^ mg protein^−1^ for FUM.

**b:** α-ketoglutarate and glyoxylate levels were measured using DNPH at 450 nm. 100% corresponds to 0.134±0.033 µmol x min^−1^ mg protein^−1^ for NAD-ICDH, 0.034±0.013 µmol x min^−1^ mg protein^−1^ for KGDH, 0.011±0.002 µmol x min^−1^ mg protein^−1^ for ICL, and 0.120±0.04 µmol x min^−1^ mg protein^−1^for MS.

**c:** activity monitored at 600 nm using DCIP. 100% corresponds to 51±4.1 nmol x min^−1^ mg protein^−1^.

**d:** Activity was assessed by quantifying BN-gel activity bands using SCION imaging. 100% corresponds to 5528 arbitrary units for complex I and 20392 arbitrary units for complex IV.

### Acylating glyoxylate dehydrogenase (AGODH) is required for oxalogenesis

**Figure 2:**
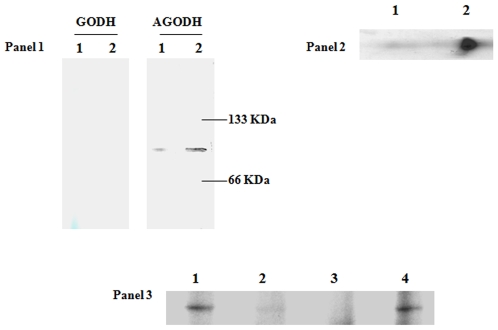
Electrophoretic analysis of enzymatic activities in *P.fluorescens*. Panel 1: In-gel activity analysis for GODH and AGODH in *Lane 1*: control, *Lane 2*: Al-stressed conditions. Panel 2: 2D SDS-PAGE analysis of the expression of AGODH in *P. fluorescens* exposed to *Lane 1*: control, *Lane 2*: Al-stressed conditions. Activity bands from Panel 1 were excised, and electrophoresed under denaturing conditions. Panel 3: Regulation of AGODH activity. *Lane 1*: Al-citrate medium; *Lane 2*: Al-stressed cells grown in a citrate medium for 18 h; *Lane 3*: citrate medium; *Lane 4*: citrate medium cells transferred to an Al-citrate medium for 18 h. Control cells were isolated at 24 h and Al-stressed cells were isolated at 28 h, respectively.

We have previously demonstrated that ICL was drastically increased in cells treated with Al compared to control. However, the increased activity of ICL was not coupled to the concomitant increase of MS; this could account for the accumulation of glyoxylate [14]. This glyoxylate appeared to be diverted towards the production of oxalate, a moiety known to render Al innocuous [21]. The amounts of oxalate were dependent on the concentration of Al in the stressed medium [22]. Hence, the two possible enzymes involved in the oxidation of glyoxylate to oxalate, glyoxylate dehydrogenase (GODH) and AGODH, were probed by in-gel activity staining. In contrast to control cells, a more intense activity band corresponding to AGODH was observed in the Al-treated cells (Figure 2, Panel 1). No activity bands were observed for GODH in either control or Al-stressed cells. A role for the NADP-dependent glyoxylate was further supported by the observation that this enzyme did not yield any band with NAD (0.1 mM) (data not shown). 2D SDS-PAGE analysis and Coomassie staining revealed a high amount of protein associated with the AGODH activity bands (Figure 2, Panel 2). To evaluate the involvement of AGODH in the adaptive response of *P. fluorescens* to Al toxicity further, we exposed the Al-treated cells to a control medium for 18 h. Similar experiments were performed with control (citrate) cells exposed to an Al-enriched medium. Exposure of the Al-challenged cells to a control medium resulted in a dramatic decrease in the activity of AGODH (Figure 2, Panel 3). In contrast, control cells incubated in an Al-enriched medium displayed an intense activity band. Thus, AGODH may be pivotal to the adaptation of *P. fluorescens* to Al toxicity.

### Al toxicity and substrate level phosphorylation

**Figure 3:**
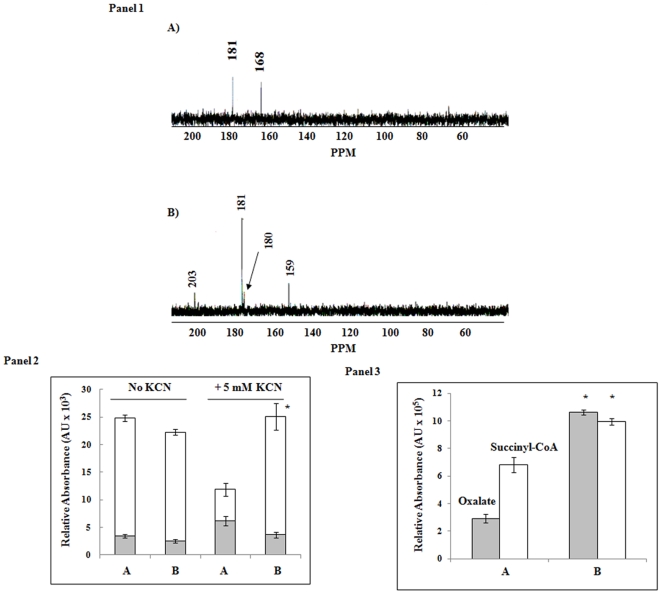
Alternative TCA cycle and substrate-level phosphorylation in *P.fluorescens*. Panel 1: Proton-decoupled ^13^C-NMR spectra obtained from the incubation of [1,4-^13^C_2_]-succinate incubated with *P. fluorescens* CFE. Panel 2: ATP production and influence of KCN. Control and Al-stressed CFE from *P.fluorescens* were incubated with 5 mM citrate, 0.5 mM ADP, and 0.1 mM CoA. ATP was measured in both the presence or absence of (5 mM) KCN. Panel 3: *P. fluorescens* CFE was incubated with 5 mM succinate, 0.5 mM NADP, 0.1 mM CoA and 5 mM glyoxylate for 1 h. Succinyl-CoA and oxalate levels were quantified using EMPOWER software. A) control and B) Al-stressed cultures. n = 3, p≤0.05, mean±S.D. * represents statistical significance in comparison to control. □ (open bar)  =  oxalate, ▪ (closed bar)  =  succinyl-CoA.

The oxidative conversion of glyoxylate to oxalate requires CoA as a cofactor with the concomitant formation of a high energy intermediate [23]. The transfer of CoA from high-energy acyl-CoAs to succinate by SCS is often coupled to the formation of ATP. In this instance, succinate is amply available due to the increased activity of ICL and the perturbation of SDH in the Al-stressed cells. In an effort to decipher how glyoxylate oxidation leads to ATP formation, the CFE from the control and Al-stressed cells were incubated with glyoxylate, ^13^C-labelled succinate, CoA, and NADP for 1 h. In the CFE obtained from the Al-stressed cells, signals at 159, 180, 181, and 203 ppm. The latter peak is indicative of the carbon in the thioester group from succinyl-CoA (Figure 3, Panel 1). In contrast, the succinate peak (181 ppm) was metabolized rapidly in the control CFE. Incubation of control and Al-stressed CFE in citrate, CoA, NADP, and ADP for 1 h in the presence and absence of KCN clearly revealed that the modified TCA cycle was responsible for the production of ATP via substrate level phosphorylation. While there was a drastic change in the ATP producing profile in the control CFE in the presence of KCN, in the stressed CFE, ATP levels did not change significantly (Figure 3, Panel 2). The accumulation of succinyl-CoA and oxalate when the CFE from the Al-stressed cells incubated with glyoxylate and succinate, clearly pointed to an important role of succinyl-CoA in substrate-level phosphorylation (Figure 3, Panel 3).

**Figure 4:**
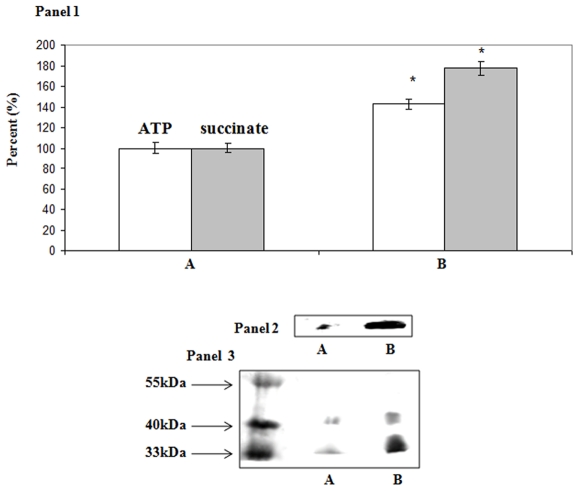
The monitoring of SCS activity and expression in *P.fluorescens*. Panel 1: HPLC analysis of ATP production by SCS. The membrane fractions from A) control and B) Al-stressed cells were incubated for 60 min in a phosphate reaction buffer containing ADP and succinyl-CoA. ATP (white bars) and succinate (gray bars) levels were quantified using EMPOWER software. The integration of control was considered to be 100% (ATP  = 2716×10^4^ and succinate  = 194×10^4^ arbitrary units). ATP and succinate were identified by injecting known standards. n = 3, p≤0.05, mean±S.D. Panel 2: In-gel activity detection of SCS. Panel 3: Bands were precision excised from Panel 1, and analyzed by SDS-PAGE. A) control and B) Al-stressed cells.

**Figure 5:**
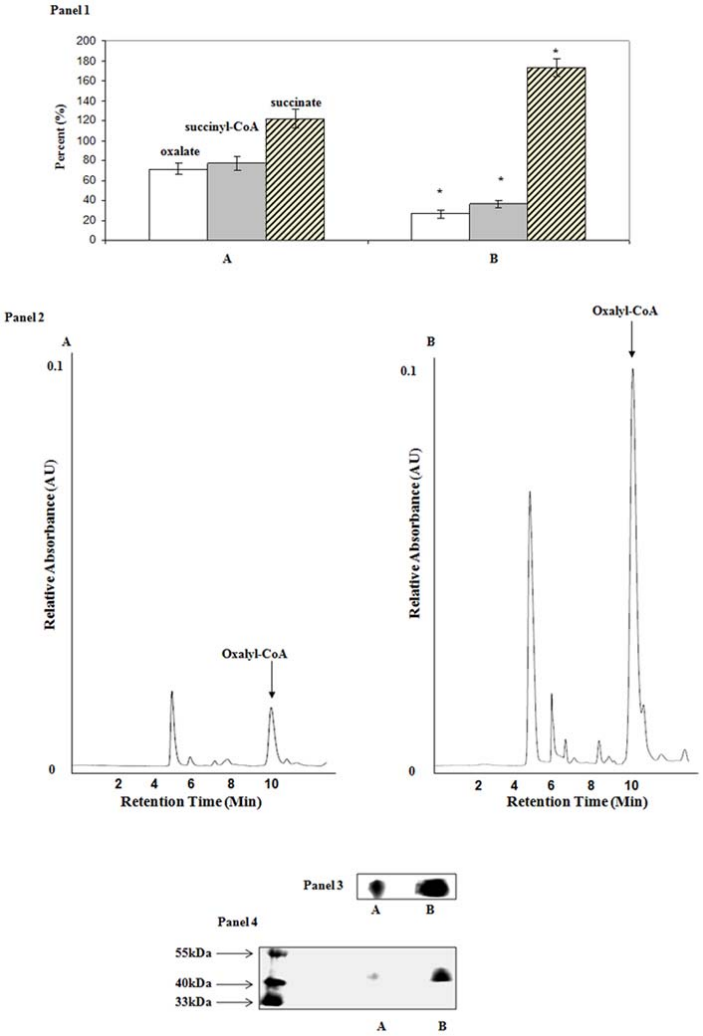
The monitoring of OCT activity and expression in *P.fluorescens*. Panel 1: HPLC analysis of OCT activity. CFE was incubated in a reaction buffer containing 0.1 mM succinyl-CoA and 0.1 mM oxalate for 5 minutes and the levels of oxalate (white bars), succinyl-CoA (gray bars), and succinate (crossed-bars) were measured. EMPOWER software was utilized to quantify the metabolites. The integration value at time  = 0 of the reaction was considered 100% (oxalate  = 16732, succinyl-CoA  = 6895, and succinate  = 14923 arbitray units, respectively). Succinate, oxalate, and succinyl-CoA were identified by injecting known standards. n = 3, p≤0.05, mean±S.D. Panel 2: Assessment of oxalyl-CoA levels. CFE was incubated in a reaction buffer containing 0.5 mM succinyl-CoA and 1 mM oxalate for 15 min. Oxalyl-CoA was identified by detecting oxalate and CoA following the treatment of the collected peak at 10 min with concentrated KOH. * represents statistical significance in comparison to control. Panel 3: In-gel activity of OCT. Panel 4: Bands were precision excised from Panel 5, and analyzed by SDS-PAGE. A) control and B) Al-stressed cells.

To verify the involvement of succinyl-CoA in ATP production, the enzyme SCS was probed. The activity of this enzyme was significantly higher in the Al-stressed cells (Figure 4, Panel 1). More ATP production was observed. In gel enzyme activity revealed a more prominent band in the CFE from the Al-stressed cells (Figure 4, Panel 2). 2D SDS-PAGE analysis aided in establishing that the level of this enzyme was elevated in the stressed-cells (Figure 4, Panel 3) [24]. Indeed, HPLC analysis pointed to the enhanced production of ATP by this enzyme in contrast to control cells (Figure 4, Panel 1). To probe the nature of this ATP-producing mechanism further, we tested the activity of OCT, a key enzyme required for the production succinyl-CoA from oxalyl-CoA [25]. A significant increase in the activity of OCT was observed in the Al-treated cells (Figure 5, Panel 1). The CFE from the Al-exposed cells also produced a major peak at 10 min that was attributed to oxalyl-CoA (Figure 5, Panel 2). This moiety readily yielded oxalate and CoA upon hydrolysis (data not shown). The in-gel activity assay and 2D SDS-PAGE helped confirm the overexpression of this enzyme in the Al-stressed cells (Figure 5, Panel 3 and 4) [26]. As Al is known to interfere with Fe metabolism, a key effector of heme biosynthesis, it was important to evaluate if the production of this metabolite was affected [8]. A drastic decrease in this moiety was observed in Al-stressed cells. Indeed, in control cells, the level of heme was 14.18 µg/mg of protein ±1.26 in the membrane fraction and 7.61μg/mg of protein ±0.15 in the cytosol. In contrast, the Al-stressed cells contained 5.64μg/mg of protein ±0.26 in the membrane fraction and 5.98μg/mg of protein ±0.53 in the cytosolic fraction.

## Discussion

The data in this study point to an alternative TCA cycle that *P.fluorescens* invokes in an effort to fulfill its fluctuating metabolic obligations in an Al-containing environment. In this instance, two moieties namely oxalate and ATP that are critical to the survival of this microbe were essentially generated via an alternative TCA cycle. Oxalate is involved in the immobilization of Al [22]. As oxidative phosphorylation was impeded, the ATP budget was augmented by enhanced substrate-level phosphorylation. The latter aspect is very important as under Al-stress, oxidative phosphorylation was sharply reduced due to dysfunctional Fe metabolism. Furthermore, citrate the sole carbon source cannot effectively provide ATP via glycolysis. ATP, the universal energy currency, can be generated by substrate-level phosphorylation and oxidative phosphorylation in aerobic organisms.

Glycolysis and the TCA cycle are two metabolic networks that can produce ATP via substrate-level phosphorylation in *P. fluorescens*. Pyruvate kinase and phosphoglycerate kinase are two glycolytic enzymes that contribute to ATP formation during hypoxia [27]. In fact, numerous cellular systems rely on glycolysis to fulfill their energy requirement. The TCA cycle can also produce ATP by substrate-level phosphorylation, a process mediated by SCS. The microbe *Trypanosoma brucei*, which is responsible for human sleeping sickness, does invoke an acetate succinate CoA transferase/succinyl CoA synthetase cycle to generate ATP. The transfer of CoA to succinate from acetyl-CoA helps preserve the energy in the thioester bond of succinyl-CoA. The succinyl-CoA is subsequently utilized to generate ATP via the phosphorylation of ADP, a process that is effected by SCS [28]. In eukaryotes, it appears that SCS exists in two isoforms. One isoform utilizes ADP as the substrate with the concomitant formation of ATP, while the other generates GTP from GDP [29]–[31]. It has recently been demonstrated that an ATP synthase deficient organism can survive by augmenting its ATP production via the substrate-level phosphorylation associated with the TCA cycle [32].

The data in the present report point to a pivotal role of SCS in ATP production. Incubation of glyoxylate, succinate, CoA, ADP with the cell-free extract of the Al-stressed cells led to a drastic increase in ATP production. In addition, high levels of oxalate were observed within the Al-exposed cells. Furthermore, an increase in OCT activity was recorded in the organism exposed to Al. Hence, SCS and OCT partner to generate ATP in the Al-stressed *P. fluorescens*. This is in sharp contrast to the downregulation of KGDH, the mediator of succinyl-CoA formation in a normally functioning TCA cycle [6]. This metabolic reorganisation will undoubtedly allow the organism to sequester Al, generate the anti-oxidant NADPH and produce ATP in an oxygen independent manner. The latter point is extremely important as Al toxicity is known to interfere with Fe metabolism and to drastically reduce the biogenesis of the respiratory complexes [8]. And as citrate is the only carbon nutrient available, the variant TCA cycle may be the only alternative route to ATP production. Hence, the upregulation of SCS will provide an evolutionary advantage to survive in an environment with limited access to oxygen. Furthermore, the upregulation of SCS will help mitigate, albeit partially, the drastic diminution of KGDH activity. From an evolutionary viewpoint, it may be unwise to completely eliminate a metabolic pathway due to a sporadic environmental flux. Thus, it is quite conceivable that the upregulation of SCS may help in this regard.

The utilization of succinyl-CoA in the generation of ATP via substrate-level phosphorylation may be triggered by the inability of the organism to effectively transport e^-^ via the respiratory complexes. These moieties contain heme. However, Al is known to trigger an Fe-deficient situation. This will render the biosynthesis of heme futile [33], [34]. Indeed, a change in the cellular proteome, in an effort to preserve Fe under Fe-limited conditions has recently been shown [35]. The lack of heme will have a major impact on the downstream processes involving the biogenesis of heme-rich proteins like complex I, II, III and IV. This will undoubtedly perturb oxidative phosphorylation and hence the stimulation of substrate-level phosphorylation associated with the TCA cycle will help compensate for this shortcoming. Furthermore, heme production in *Pseudomonas* has been shown to utilize glutamate and NADPH [36], [37]. In Al-stressed *P.fluorescens*, these metabolites may be diverted in an effort to combat oxidative stress. While NADPH is critical to the proper functioning of numerous anti-oxidative enzymes, glutamate has been shown to generate KG, a potent ROS scavenger [7], [38]. This situation may contribute to the decreased heme synthesis observed in the Al-stressed conditions. Hence, enhanced TCA-cycle mediated substrate-level phosphorylation may help signal a dysfunctional Fe-metabolism and evoke a response reminiscent of anaerobiosis [39]. Therefore, the conversion of succinyl-CoA into ATP may be an important metabolic adaptation under Fe-limitation and O_2_-deficient conditions, two situations inherent of Al toxicity. The significance of the TCA cycle in metabolic adaptation and as a signaling network is only now beginning to emerge [40], [41]. As in most organisms, succinyl-CoA is a participant in heme synthesis, it is tempting to postulate that the diversion of this high energy metabolite may be involved in signalling anaerobic situations [42].

The formation of oxalate via a reconfigured TCA cycle may also help the microbe immobilize the toxic metal. It is tempting to postulate that the downregulation of NAD-dependent ICDH and KGDH allows this organism to create a TCA cycle that instead of evolving 2CO_2_ from acetyl CoA, transforms this moiety into oxalate. The net effect of this metabolic adaptation is in fact a TCA cycle with limited CO_2_ release, diminished NADH formation and increased production of oxalate, NADPH and ATP. This is a classical example of an organism utilizing pre-existing biochemical reactions to create a novel metabolic pathway aimed at circumventing an environmental challenge. This adaptation provides a glimpse into how metabolic networks may have evolved. Oxalate sequesters Al and NADPH guards against Al-induced ROS. ATP obtained via substrate-level phosphorylation helps the organism survive an anaerobic condition promoted by Al. It is also important to note that ICL and MS that usually work in partnership as part of the glyoxylate cycle were uncoupled in this study [43]. In the Al-stressed cells, ICL activity was elevated while the activity of MS was relatively unchanged. This modification will allow the channeling of glyoxylate towards the biogenesis of oxalate, a metabolite utilized in the sequestration of Al [21], [22]. It is noteworthy to mention that upregulation of fumarase C, an Fe-independent enzyme recently uncovered in Al-stressed *P. fluorescens* may help provide an alternate route to malate than the one mediated by MS [44].

Since Al has been found to create an intracellular oxidative environment, it is not surprising that NADPH-generating enzymes are markedly increased. We have shown the increment in both activity and expression of such enzymes as glucose-6-phosphate dehydrogenase, NADP-dependent ICDH and malic enzyme (ME) in *P. fluorescens* subjected to Al toxicity [45]. The NADPH-generating systems are known to help guard enzymes involved in the direct disposal of such ROS as O_2_^−^ and H_2_O_2_. Thus, as the demand for NADPH is accentuated during Al-stress, it is not surprising that upregulation of AGODH may contribute to the NADPH budget and the formation of oxalyl-CoA. The energy stored in this thioester bond is eventually transferred to succinate with the formation of the energy-rich succinyl-CoA. Succinate is readily available during Al stress due to the upregulation of ICL. The increased activity of SCS will help phosphorylate ADP into ATP with the participation of succinyl-CoA Hence, this metabolic arrangement allows the production of three products namely oxalate, NADPH and ATP all important guardians against Al toxicity.

**Figure 6:**
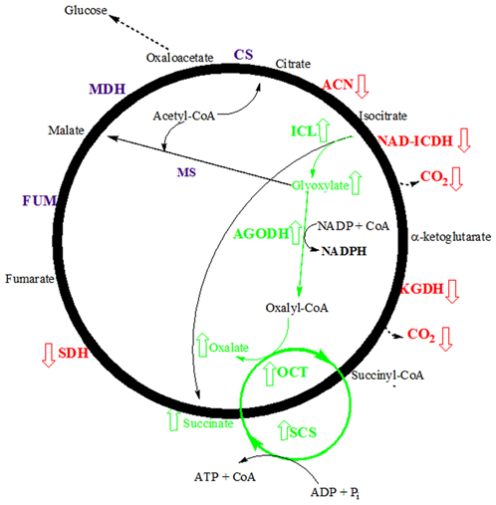
An alternative TCA cycle generates oxalate and ATP as a consequence of Al toxicity. (↓) represents a decrease in enzyme activity whereas (↑) represents an increase in activity. AGODH  =  acylating glyoxylate dehydrogenase, SCS  =  succinyl-CoA synthetase, ICL  =  isocitrate lyase, MS  =  malate synthase, ACN  =  aconitase, NAD-ICDH  =  NAD-dependent isocitrate dehydrogenase, SDH  =  succinate dehydrogenase, FUM  =  fumarase, OCT  =  oxalate CoA-transferase, and KGDH  =  α-ketoglutarate dehydrogenase, CS  =  citrate synthase, MDH  =  malate dehydrogenase.

In conclusion, the present study shows how this alternative TCA cycle enables *P.fluorescens* to generate the three critical metabolites (oxalate, ATP, and NADPH) with the concomitant reduction in the synthesis of NADH. This fine metabolic-balancing act is crucial for the survival of the microbe. In this instance, succinyl-CoA is key to the adaptation to Al toxicity as it helps generate ATP. The flux in the concentration of succinyl-CoA may indeed reflect the status of the TCA cycle, cellular O_2_ gradient and Fe homeostasis. It is also important to note that this variant TCA cycle instead of releasing CO_2_ from acetyl-CoA fixes it into oxalate. Figure 6 depicts a scheme that illustrates the alternative TCA cycle evoked by Al-toxicity.
